# Characteristics of Facial Muscle Activity Intensity in Patients With Schizophrenia and Its Relationship to Negative Symptoms

**DOI:** 10.3389/fpsyt.2022.829363

**Published:** 2022-02-21

**Authors:** Xia Du, Hong Zhen Fan, Yun Hui Wang, Jie Zhang, Xiao Lin Zhu, Yan Li Zhao, Shu Ping Tan

**Affiliations:** Beijing HuiLongGuan Hospital, Beijing, China

**Keywords:** schizophrenia, facial muscles, activity intensity, negative symptoms, emotional stimuli

## Abstract

**Introduction:**

Previous studies have shown that in addition to having impairments in facial emotion recognition, patients with schizophrenia also show a lack of facial expression. Although negative symptoms such as decreased facial activity are common symptoms of schizophrenia, the related factors remain inconclusive. Therefore, this study compared healthy controls to explore the characteristics of facial muscle activity intensity in patients with schizophrenia and its relationship with negative symptoms.

**Methods:**

This observational and cross-sectional study conducted in a psychiatric hospital in China included a total of 135 patients with schizophrenia and 134 healthy controls. The negative symptoms of schizophrenia were evaluated using the Brief Negative Symptom Scale. The intensity of facial muscle activity under positive, neutral, and negative emotional stimuli conditions was automatically collected by a computer, including 17 values (F01-F17) that represent different facial muscle activities. Statistical tests were performed to analyze facial muscle activity indexes, to explore an objective and quantitative method to evaluate the negative symptoms of schizophrenia.

**Results:**

The facial muscle activity intensity of the schizophrenia group at F02 (outer eyebrow), F04 (upper eyelid), F07 (nose), F10 (dimple), F12 (lower jaw 1), F14 (lip 2), and F17 (blink) was lower than that of the healthy controls (*p* < 0.05). Under positive, neutral, and negative emotional stimuli conditions, the facial muscle activity intensity of F16 (lower jaw 2) was positively correlated with negative symptoms (*p* < 0.05).

**Conclusion:**

Our study indicated that patients with schizophrenia show defects in facial muscle activity and that is associated with negative symptoms.

## Introduction

Schizophrenia is a serious mental disorder. According to an epidemiological survey in China in 2019, the lifetime prevalence of schizophrenia is 0.6%, second only to depressive and anxiety disorders (1). Moreover, surveys in some cities in China show that schizophrenia accounts for 70.8% of psychiatric diseases and 46.7% of hospitalized patients and that these figures trend upward annually (2, 3). Therefore, schizophrenia has placed a serious burden on Chinese patients' families and society. The common symptoms of schizophrenia include positive and negative symptoms; negative symptoms such as decreased facial activity and indifference are the most common initial symptoms of schizophrenia (4). Negative symptoms exist even in the early stages of the disease, but they are easily masked by strong positive symptoms, so they may be delayed and even gradually aggravated, which is also an important reason for the high disability rate in relation to schizophrenia. At present, the primary methods of evaluation for negative symptoms either are various scales or rely on the subjective judgment of doctors' clinical experience, and there is a lack of objective and quantitative evaluation methods.

The existence of negative symptoms may also lead to cognitive impairment (5, 6), especially social cognitive impairment with emotional expression as the core. A study on the autonomous facial emotion expression of patients with schizophrenia (that is, in the absence of stimulation materials, subjects spontaneously express facial emotions such as sadness, anger, and happiness) suggests that the emotional expression ability of such patients is significantly weaker than that of healthy controls; moreover, the impairment of emotional expression is related to the score of negative symptoms (7). In another study, in daily life, patients with schizophrenia talked with their families about numerous practical problems. The study found that the number of facial expression changes in patients with schizophrenia per unit time was significantly lower than that in healthy controls, and the correlation between facial expression changes and conversation content decreased (8). It is suggested that there are obvious defects in both intentional emotional expression and autonomous emotional expression in patients with schizophrenia. These defects are not caused by clinical symptoms and drug side effects, but rather by a group of independent symptoms (9). Although the above studies suggest that there is a correlation between facial emotional expression and the negative symptoms of schizophrenia, these studies either only focus on a designated emotional expression or only study the indicators of the number of changes in facial expression and cannot completely quantitatively evaluate negative symptoms.

Therefore, we assume that the facial muscle activity of patients with schizophrenia under the same emotional stimuli is different from that of healthy controls, which may reflect the severity of negative symptoms. We preliminarily explore negative symptoms using an objective and quantitative evaluation method to provide reference for the symptom evaluation of auxiliary diseases. This will assist clinicians in noticing negative symptoms earlier, assessing the severity of negative symptoms more accurately, and formulating intervention plans for negative symptoms earlier so as to reduce the social function defects caused by negative symptoms as well as to reduce the burden on patients' families and society.

## Materials and Methods

### Subjects

Based on the previous literature on the changes of facial emotion in relation to the negative symptoms of schizophrenia, the reported effect value is 0.80 (10), calculated by G-Power software using a two-tailed test and setting α = 0.05 and 1- β = 0.85; the ratio between the case group and the control group is set to 1:1, and the sample size of each group is 30 cases. The subjects were outpatients and inpatients with schizophrenia at Beijing HuiLongGuan Hospital between October 2017 and July 2019. Healthy controls were recruited through the community and media advertising during the same period. The inclusion criteria for patients with schizophrenia were as follows: meeting the diagnostic criteria for schizophrenia in the Diagnostic and Statistical Manual of Mental Disorders Fourth Edition (DSM-IV) (11), being aged 18–60 years old, and having a junior high school level of education or above. Exclusion criteria were having an intellectual disability, having a serious physical diseases or adverse drug reactions, substance dependence or abuse, having excited, impulsive behavior or, during the decline associated with schizophrenia, being unable to cooperate with the test, and lactation or pregnancy. Inclusion criteria for healthy controls were as follows: having no abnormal mental state in the fixed interview with psychiatrists, having no family history of mental disorders, being aged 18–60 years old, and having a junior high school level of education or above. Exclusion criteria were having an intellectual disability, suffering from serious physical diseases, substance dependence or abuse, and lactation or pregnancy. Following the application of the inclusion and exclusion criteria, a total of 160 patients with schizophrenia and 143 healthy controls were included. Because some subjects failed to complete the experimental task, 135 patients with schizophrenia and 134 healthy controls were ultimately included in the study. The task comprised measurements of facial muscle strength under three different emotional states as well as evaluation of the scale to determine whether there is a difference in the intensity of facial muscle activity between the two groups and whether the difference correlates with negative symptoms of schizophrenia.

This study was approved by the ethics committee of Beijing HuiLongGuan Hospital. After the subjects were fully informed of the study plan, we obtained their written informed consent to participate in the study.

### Basic Information and Clinical Symptom Evaluation

The gender, age, and years of education of the subjects were collected using a self-made basic information questionnaire. The Brief Negative Symptom Scale (BNSS) (12) was used to evaluate the severity of negative symptoms in patients with schizophrenia. There are 13 items on the scale, including six subscales: anhedonia subscale, depression subscale, blunted affect subscale and so on. Each of the 13 items is rated on a 7-point scale (0–6). The total score ranges from 0–78, with a higher score indicating more serious symptoms. The reliability of the scale was evaluated by psychiatrists who had received unified training, and the consistency among raters was good (intra-class correlation coefficient, *ICC* > 0.8).

### Evaluation of Facial Muscle Activity Intensity (FMAI)

**Figure 1:**
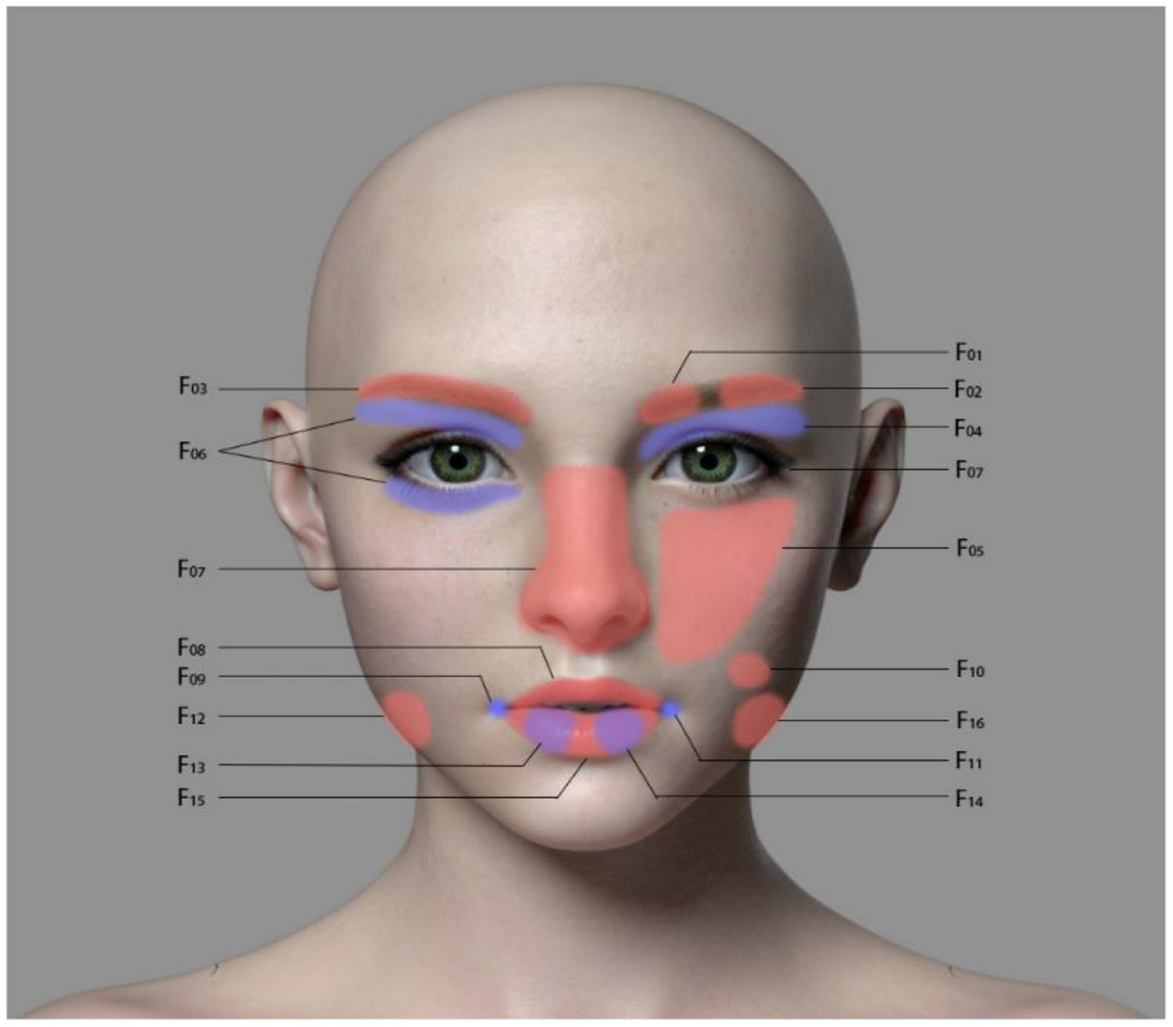
The 17 sites of facial muscle activity intensity measurement in this study.

The subjects sat in front of a computer with a camera (full HD camera, model Logitech C920 Pro) facing them about 80cm from their face, such that they subject could watch (as well as hear the corresponding audio) three videos including positive, neutral, and negative emotions, respectively. The name of the positive-emotion video is “Funny insects”; this video depicts interesting things that happen to three insects together, and the plot is humorous. The neutral-emotion video is called “The millennium of the universe” and is a documentary about astronauts exploring the universe. Finally, the negative-emotion video is called “Besieged city in October” and describes an old father's grief-stricken scene after seeing his son killed. The computer automatically collected the facial muscle activity state of the subjects throughout the entire experiment under the projection of the three emotional stimuli. The program E-face was used to process the data related to facial muscle activity. The duration of the positive, neutral, and negative videos was 83 s, 82 s, and 95 s, respectively. The corresponding facial muscle activity data were intercepted according to the time start and end points with a sampling rate was 20 frames per second. For each subject, the total sampling rate of positive, neutral, and negative stimuli was approximately 1,660, 1,620, and 1,900 frames, respectively. Each frame collects 17 values (F01-F17), representing the intensity of 17 facial muscle activities. The data collection range was divided according to Ekman's facial action coding system (FACS) (13), which included the following, as shown in Figure 1: F01, inner eyebrow; F02, outer eyebrow; F03, eyebrow; F04, upper eyelid; F05, cheek; F06, eyelid; F07, nose; F08, upper lip; F09, lip angle 1; F10, dimple; F11, lip angle 2; F12, lower jaw 1; F13, lip 1; F14, lip 2; F15, lip 3; F16, lower jaw 2; and F17, blink.

### Statistical Analysis

The Statistical Package for the Social Sciences (SPSS) version 20.0 (IBM, USA) was used for data analysis. The chi-square test was used for comparison between continuous data groups. Repeated measurement analysis of variance (ANOVA) was used to test the intensity of the facial muscle activity of the two groups (schizophrenia group, SZ; healthy control group, HC) under three different emotional stimuli conditions, used Bonferroni correction (statistical significance set at *p* < 0.003). Taking the patient group and the healthy control group as dependent variables and the facial muscle activity intensity of different parts as independent variables, F01-F17 facial muscle activity intensity was included in the logistic regression model for logistic regression analysis. Taking the total BNSS score as the dependent variable and the intensity of facial muscle activity under three emotional stimuli conditions as the independent variable, the relationship between facial muscle activity intensity and negative symptoms was analysed by multiple linear regression. Statistical significance was set at *p* < 0.05.

## Results

### Demographic and Clinical Characteristics

There was no significant difference in sex and age between the schizophrenia group (*n* = 135) and healthy control group (*n* = 134) (*p* > 0.05), but there was a significant difference in the years of education between the two groups (*p* < 0.05) in that the schizophrenia group had fewer years of education than the healthy control group (Table 1).

**Table 1:** Demographic and clinical data for the schizophrenia group and the healthy control group (*N* = 269).

	**SZ (*n* = 135)**	**HC (*n* = 134)**	***t*/χ^2^**	***p***
Gender, *n* (%)			3.57	0.059
Male	76 (56.30)	60 (44.78)		
Female	59 (43.70)	74 (55.22)		
Age (years) (Mean ± SD)	43.28 ± 11.54	41.32 ± 11.13	−1.42	0.157
Education (years) (Mean ± SD)	13.07 ± 2.57	14.21 ± 2.95	3.36	0.001^**^
Course of disease (years) (Mean ± SD)	11.69 ± 9.32			
BNSS total score (Mean ± SD)	23.79 ± 13.49			

** *p < 0.01*.

*SZ, schizophrenia group; HC, healthy control*.

### Facial Muscle Activity Intensity Under Different Emotional Stimuli Conditions

A comparison of facial muscle activity intensity between the schizophrenia group and the healthy control group under three different emotional stimuli conditions is outlined in Table 2. Repeated measurement ANOVA showed that the grouping main effect of F11, F13, and F17 facial muscle activity intensity was statistically significant (*p* < 0.003), while the emotional main effect of F03, F05, F09, F11, F12, F15, and F17 facial muscle activity intensity was statistical significance (*p* < 0.003). The interaction effect of grouping and emotion of F05, F06, F08, F09, and F10 was statistically significant (*p* < 0.003).

**Table 2:** Comparison of facial muscle activity intensity between the schizophrenia group and the healthy control group under three different emotional stimuli conditions.

**Face Muscle**	**SZ (Mean** **±SD)**		**HC (Mean** **±SD)**				**Group**		**Emotion**		**Group** **× Emotion**	
	**PSE**	**NUE**	**NGE**	**PSE**	**NUE**	**NGE**	**F**	**p**	**F**	**p**	**F**	**p**
F01	1.79 ± 1.45	1.79 ± 1.44	1.87 ± 1.45	1.34 ± 1.41	1.61 ± 1.30	1.65 ± 1.39	3.42	0.065	5.15	0.007	2.75	0.069
F02	1.72 ± 1.35	1.71 ± 1.38	1.78 ± 1.39	1.45 ± 1.21	1.64 ± 1.11	1.75 ± 1.16	0.78	0.377	4.88	0.009	2.60	0.078
F03	1.00 ± 0.76	1.03 ± 0.81	1.12 ± 0.81	0.88 ± 0.79	0.89 ± 0.77	0.99 ± 0.79	2.12	0.147	8.44	0.000^*^	0.16	0.843
F04	1.18 ± 0.75	1.16 ± 0.75	1.19 ± 0.82	1.07 ± 0.74	1.21 ± 0.75	1.28 ± 0.83	0.02	0.902	5.98	0.004	5.91	0.004
F05	0.74 ± 0.61	0.75 ± 0.61	0.76 ± 0.59	0.82 ± 0.58	0.62 ± 0.52	0.59 ± 0.55	1.17	0.280	13.96	0.000^*^	20.19	0.000^*^
F06	1.24 ± 0.87	1.26 ± 0.89	1.28 ± 0.95	1.17 ± 0.84	1.01 ± 0.82	0.97 ± 0.85	4.31	0.039	4.35	0.014	8.47	0.000^*^
F07	0.90 ± 0.66	0.95 ± 0.73	0.96 ± 0.74	0.84 ± 0.66	0.69 ± 0.62	0.71 ± 0.70	6.12	0.014	1.19	0.304	5.97	0.004
F08	0.92 ± 0.62	0.96 ± 0.61	0.98 ± 0.67	0.96 ± 0.63	0.77 ± 0.60	0.85 ± 0.65	1.72	0.191	3.91	0.023	9.88	0.000^*^
F09	0.65 ± 0.50	0.63 ± 0.49	0.61 ± 0.48	0.71 ± 0.45	0.53 ± 0.38	0.51 ± 0.41	0.84	0.360	21.13	0.000^*^	9.65	0.000^*^
F10	0.88 ± 0.63	0.96 ± 0.60	0.92 ± 0.63	1.04 ± 0.71	0.87 ± 0.75	0.86 ± 0.69	0.00	0.987	2.64	0.075	10.15	0.000^*^
F11	1.81 ± 1.28	1.93 ± 1.17	2.01 ± 1.23	1.18 ± 0.88	1.50 ± 1.01	1.45 ± 0.95	18.72	0.000^*^	19.10	0.000^*^	2.52	0.081
F12	1.26 ± 0.93	1.41 ± 0.91	1.48 ± 0.93	1.43 ± 0.95	1.64 ± 0.89	1.66 ± 0.94	3.56	0.060	20.21	0.000^*^	0.39	0.663
F13	1.18 ± 0.79	1.21 ± 0.74	1.24 ± 0.79	0.86 ± 0.54	0.84 ± 0.52	0.89 ± 0.52	23.58	0.000^*^	1.19	0.303	0.51	0.593
F14	1.15 ± 0.82	1.31 ± 0.81	1.32 ± 0.85	1.14 ± 0.68	1.15 ± 0.70	1.24 ± 0.73	1.07	0.302	5.95	0.003	1.67	0.190
F15	1.10 ± 0.78	1.02 ± 0.81	1.06 ± 0.79	1.19 ± 0.95	0.96 ± 0.79	0.93 ± 0.80	0.13	0.723	9.49	0.000^*^	3.82	0.026
F16	1.32 ± 0.99	1.35 ± 1.00	1.33 ± 1.08	1.37 ± 1.00	1.26 ± 1.02	1.33 ± 1.06	0.01	0.910	0.34	0.706	1.49	0.228
F17	1.18 ± 0.88	1.31 ± 0.80	1.34 ± 0.84	0.88 ± 0.72	0.96 ± 0.71	0.95 ± 0.76	15.47	0.000^*^	7.83	0.001^*^	1.04	0.353

* *p < 0.003*.

*SZ, schizophrenia group; HC, healthy controls; PSE, positive emotion; NUE, neutral emotion; NGE, negative emotion*.

The facial muscle activity intensity of the schizophrenia group in F11, F13, and F17 was higher than that of the healthy control group. The intensity of facial muscle activity for positive emotion was higher than that of neutral and negative emotion at F05, F09 and F15, and lower at F11 and F17; the facial muscle activity intensity of negative emotion was higher than that of positive and neutral emotion at F03. Further simple effect analysis of the inteaction showed that, the intensity of facial muscle activity for positive emotion was higher than that of neutral and negative emotion at F05, F06, F08, F09 and F10 in healthy control group, and was not significant in patient group (*p* < 0.05).

### Facial Muscle Activity Intensity in the Schizophrenia and Healthy Control Groups

Taking the grouping of the schizophrenia group and healthy control group as dependent variables and the intensity of facial muscle activity in different parts of the face as independent variables, all intensities of facial muscle activity for F01-F17 were simultaneously included in the logistic regression model. The results of the logistic regression analysis showed that facial muscle activity for F02, F04, F07, F10, F12, F14, and F17 was significantly different between the schizophrenia and healthy control groups (*p* < 0.05). The accuracy of the logistic regression model was 69.6% for predicting schizophrenia, 77.6% for predicting healthy controls, and 73.6% in total (Table 3).

**Table 3:** Results of the logistic regression model of facial muscle activity intensity in the schizophrenia group and the healthy control group.

**Independent variable**	**β**	***SE***	***Wals***	***df***	***p***	***Exp (B)***	***95%CI***
F01	0.23	0.18	0.02	1	0.902	1.02	1.48–1.80
F02	0.42	0.13	10.07	1	0.002^**^	1.52	1.50–1.77
F03	−0.25	0.25	0.97	1	0.325	0.78	0.79–0.95
F04	0.55	0.21	7.14	1	0.008^**^	1.73	1.12–1.30
F05	−0.55	0.41	1.86	1	0.172	0.58	0.69–0.82
F06	0.31	0.24	1.58	1	0.208	1.36	1.10–1.29
F07	0.79	0.25	10.04	1	0.002^**^	2.20	0.75–0.90
F08	0.18	0.37	0.24	1	0.627	1.20	0.76–0.88
F09	−0.41	0.44	0.85	1	0.358	0.67	0.59–0.68
F10	−0.58	0.25	5.44	1	0.020^*^	0.56	0.93–1.07
F11	0.14	0.19	0.60	1	0.437	1.16	1.30–1.53
F12	−0.64	0.19	11.19	1	0.001^**^	0.53	1.34–1.55
F13	0.44	0.32	1.86	1	0.172	1.55	0.91–1.04
F14	0.62	0.24	6.48	1	0.011^*^	1.85	1.10–1.26
F15	0.04	0.29	0.21	1	0.885	1.04	1.04–1.20
F16	−0.17	0.22	0.62	1	0.433	0.84	1.31–1.55
F17	0.63	0.21	9.26	1	0.002^**^	1.88	0.92–1.10

* *p < 0.05*;

** *p < 0.01*.

The total score of BNSS was taken as the dependent variable, and the intensity of facial muscle activity was taken as the independent variable; α = 0.05, excluding level β = 0.10. The results of multiple linear regression analysis using the enter method showed that the variables that were significant for negative symptoms were screened. Under positive, neutral, and negative emotional stimulation, the intensity of F16 facial muscle activity in patients was positively correlated with the total score of BNSS, and the model was statistically significant (*p* < 0.05). Positive emotion F12, neutral emotion F16, negative emotion F16 facial muscle activity intensity was positively correlated with the score of Anhedonia subscale; Neutral emotion F12 facial muscle activity intensity was positively correlated with the score of Depression subscale; Positive, neutral and negative emotion F16 facial muscle activity intensity was positively correlated with the score of Asociality subscale and Avolition subscale; Positive emotion F01 and F07, neutral emotion F15 facial muscle activity intensity was positively correlated with the score of Blunted affect subscale. This correlation was not statistically significant in other facial muscle activity intensity models (*p* > 0.05) (Table 4). See the Supplementary Material for other results.

**Table 4:** Multiple linear regression analysis of facial muscle activity intensity related to negative symptoms of schizophrenia.

**Variable**	**β**	***SE***	***β'***	***t***	***p***
PSE F16	3.44	1.15	0.25	2.99	0.003^**^
NUE F16	3.07	1.14	0.23	2.70	0.008^**^
NGE F16	2.49	1.07	0.20	2.34	0.021^*^

* *p < 0.05*;

** *p < 0.01. PSE, positive emotion; NUE, neutral emotion; NGE, negative emotion*.

## Discussion

Previous studies have shown that in addition to having impairments in facial emotion recognition, patients with schizophrenia also show a lack of facial expression. This study directly measured the facial muscle activity intensity of patients with schizophrenia under different emotional stimuli conditions to explore the attributes of negative symptoms that may lead to a reduction in facial activity. Our study had two main findings. First, we found that some facial muscle activity intensity in patients with schizophrenia was worse than that in healthy controls. Second, the weakening of some facial muscle activity intensity in patients with schizophrenia may be related to negative symptoms, suggesting that schizophrenia may have some defects in the expression of basic social emotions, which provides a theoretical basis for further exploring the characteristics of schizophrenia in the expression of basic social emotions in the future.

This study showed that under the same emotional stimulation, the facial muscle activity intensity of patients with schizophrenia is lower than that of healthy controls; this suggests that this effect does not have emotional specificity, which is consistent with the results of previous studies (14–17) and may be related to face emotion recognition disorder in patients with schizophrenia. In this regard, corresponding psychological and physiological responses to different emotional stimuli cannot be fully expressed, which leads to the changes in facial muscle activities behind facial expressions. Some studies suggest that this may be related to changes in brain activation (18). Moreover, a small number of studies have reported that the facial muscle activity of patients with schizophrenia can be fully expressed (19, 20). Previous studies of basic socio-emotional perception primarily focused on facial emotion recognition and expression; however, cultural differences may affect patients in a variety of ways according to ethnicity, nationality, or race. This may be related to inconsistent measurement tools: those studies also used electromyography measurements with electrodes and sensors connected to the subject's face (21), while in this study, we used a full HD optical camera for computer automatic acquisition and measurement of facial muscle activity intensity, which is not affected by the above factors. Measurements were taken in positive, neutral, and negative emotional states rather than dichotomously divided into happiness and anger, and these were combined with clinical symptoms for effective results (22). The intensity may also be related to different stages of schizophrenia. Most of the patients in this study were long-term hospitalized patients in chronic remission. At the same time, this study also suggests that the decrease in facial muscle activity intensity in schizophrenia is related to negative symptoms, which is consistent with previous research results (4, 10). This may be related to the pathological mechanism of attention bias and memory loss related to emotional information (23) as well as to the defect of facial muscle activity caused by facial emotion recognition disorder, which is then followed by negative symptoms (24).

The findings of this study are also consistent with clinical experience; that is, patients with schizophrenia will show a decrease in overall facial muscle activity, poor richness of emotional expression, and more simplicity and repeatability, and they will exhibit basic socio-cognitive emotion impairment with negative symptoms as the core (25, 26).

This study has several limitations. First, this is a cross-sectional study, which cannot facilitate dynamic understanding of the changes of facial muscle activity over the course of the disease. Second, the patient group is still receiving drug treatment, and the effect of drugs and Parkinsonian side effects (caused by anti-psychotics) on facial muscle activity cannot be ruled out. Therefore, he current study can be viewed only as an exploratory study. In future studies, we plan to include patients at first onset or patients who are not undergoing drug treatment and to conduct follow-up studies to further understand the characteristics of facial muscle activity in patients with schizophrenia.

## Conclusions

The intensity of some facial muscle activity of patients with schizophrenia is damaged to some degree, an effect that is may be related to the negative symptoms of schizophrenia. Whether the intensity of facial muscle activity can be used as an index to evaluate the negative symptoms and severity of schizophrenia needs to be further explored in future research.

## Data Availability Statement

The original contributions presented in the study are included in the article/Supplementary Material, further inquiries can be directed to the corresponding author.

## Ethics Statement

The studies involving human participants were reviewed and approved by the Ethics Committee of Beijing HuiLongGuan Hospital. The patients/participants provided their written informed consent to participate in this study.

## Author Contributions

XD, HF, YW, JZ, XZ, YZ, and ST provided different contributions to this research, such as the collection of subjects, data sorting, and article writing guidance. All authors agreed to the publication of the article.

## Funding

This work was supported by the Beijing Natural Science Foundation (Grant Number: 7162087, 2016), Capital Clinical Characteristic Application Research (Grant Number: z141107002514016, 2014), and the National Natural Science Foundation of China (Grant Number: 31671145, 2017).

## Conflict of Interest

The authors declare that the research was conducted in the absence of any commercial or financial relationships that could be construed as a potential conflict of interest.

## Publisher's Note

All claims expressed in this article are solely those of the authors and do not necessarily represent those of their affiliated organizations, or those of the publisher, the editors and the reviewers. Any product that may be evaluated in this article, or claim that may be made by its manufacturer, is not guaranteed or endorsed by the publisher.
