# Supplementary material for: Characteristics of Facial Muscle Activity Intensity in Patients With Schizophrenia and Its Relationship to Negative Symptoms
Source: Front Psychiatry. 2022 Feb 21;13:829363. doi: 10.3389/fpsyt.2022.829363 (PMC8900141; doi:10.3389/fpsyt.2022.829363)
Supplement: Supplementary file 1 [file Table_1.docx]

**Supplementary Table 1**. Multiple linear regression analysis of facial muscle activity intensity related to negative symptoms of schizophrenia.

| **Variable** | | ***β*** | ***SE*** | ***β'*** | ***t*** | ***p*** |
| --- | --- | --- | --- | --- | --- | --- |
| Anhedonia subscale | PSE F12 | 0.87 | 3.42 | 0.22 | 2.54 | 0.012* |
|  | NUE F16 | 0.66 | 0.32 | 0.18 | 2.04 | 0.043* |
|  | NGE F16 | 0.62 | 0.30 | 0.18 | 2.07 | 0.040* |
| Depression subscale | NUE F12 | 0.30 | 0.14 | 0.19 | 2.20 | 0.030* |
| Asociality subscale | PSE F16 | 0.73 | 0.21 | 0.30 | 3.56 | 0.001** |
|  | NUE F16 | 0.69 | 0.20 | 0.29 | 3.45 | 0.001** |
|  | NGE F16 | 0.61 | 0.19 | 0.27 | 3.25 | 0.001** |
| Avolition subscale | PSE F16 | 0.66 | 0.23 | 0.25 | 2.90 | 0.004** |
|  | NUE F16 | 0.60 | 0.22 | 0.23 | 2.68 | 0.008** |
|  | NGE F16 | 0.47 | 0.21 | 0.19 | 2.24 | 0.027* |
| Blunted affect subscale | PSE F01 | 0.53 | 0.25 | 0.19 | 2.09 | 0.038* |
|  | PSE F07 | 1.42 | 0.53 | 0.24 | 2.67 | 0.009** |
|  | NUE F15 | 0.94 | 0.43 | 0.19 | 2.20 | 0.030* |

* *p* < 0.05; * * *p* < 0.01.
